# A review of the application of intelligent sensing technology in the recognition and evaluation of facial paralysis

**DOI:** 10.3389/fnins.2025.1646485

**Published:** 2025-09-12

**Authors:** Jingyao Wang, Xing Tang, Jicheng Zhang, Xin Shao, Yang Liu, Yan Zhai, Jili Xu, Shoujen Lan, Yeayin Yen, Chao Wang

**Affiliations:** ^1^Daytime Internal Medicine Treatment Area, Sichuan Cancer Hospital and Institute, Chengdu, China; ^2^College of Acupuncture-Moxibustion and Tuina, Chengdu University of Traditional Chinese Medicine, Chengdu, China; ^3^Department of Acupuncture, Sichuan Integrative Medicine Hospital, Chengdu, China; ^4^Big Data and Intelligent Equipment Research Laboratory, Sichuan Institute for Translational Chinese Medicine, Chengdu, China; ^5^Department of Healthcare Administration, Asia University, Taiwan, China; ^6^Sub-Health Clinical Medicine Research Center, Sichuan Integrative Medicine Hospital, Chengdu, China

**Keywords:** intelligent sensing technology, facial paralysis, surface electromyogram, three-dimensional motion capture, artificial intelligence

## Abstract

Facial paralysis (FP), as a highly prevalent neurological dysfunction disease worldwide, has long faced challenges such as strong subjectivity in assessment and difficulty in quantifying therapeutic effects in its clinical diagnosis and treatment. Traditional scales rely on physicians’ experience. Neuroelectrophysiological examinations are invasive, while imaging evaluations are costly. The rise of intelligent sensing technology provides a new path to break through these limitations. Intelligent sensing technology has significantly improved the accuracy of FP recognition and assessment through multi-modal data fusion and dynamic monitoring. Its clinical value is not only reflected in the improvement of diagnostic efficiency, but also in promoting a fundamental change in the diagnosis and treatment model of FP. The artificial intelligence-assisted analysis mainly focuses on using machine learning algorithms to conduct in-depth exploration and analysis of the surface electromyogram (sEMG) signals of patients with facial paralysis, the motion trajectory data obtained through three-dimensional (3D) motion capture, as well as the data from patients’ self-assessment scales. This study systematically reviews the innovative applications of intelligent sensing technology in the recognition and evaluation of FP, focusing on three major technical directions: sEMG, 3D motion capture, and artificial intelligence assisted analysis.

## 1 Introduction

Facial paralysis (FP), as a highly prevalent neurological dysfunction disease worldwide, faces severe challenges in its clinical diagnosis and treatment. According to the World Health Organization (WHO), the global annual incidence rate of FP is approximately 11.5 per 100,000 to 40.2 per 100,000 people. The high-incidence age of this disease is 15 to 45 years old. However, the incidence rate among people over 60 years old increases significantly with age, and the risk further intensifies after the age of 70 (Bruins et al., 2021). It is worth noting that the prevalence rate among women is 20% higher than that among men, especially during the reproductive period and the third trimester of pregnancy (Lin and Sol, 2020). The clinical harm of FP far exceeds the disease itself. Studies show that 85% of patients suffer from social anxiety due to facial asymmetry, 40% develop depressive tendencies, and their occupational performance scores decrease by 37% compared to healthy people (Hotton et al., 2020). Long-term FP can also cause complications such as corneal ulcers and dysphagia, significantly reducing the quality of life (Fu et al., 2011).

The traditional FP assessment system has three core flaws: Firstly, the problem of insufficient reliability and validity of subjective scales is particularly prominent. The House-Brackmann scale, which is the clinical gold standard, has an inter-evaluator consistency coefficient of only 0.77. Especially in the diagnosis of grade III - IV FP, the Kappa value is as low as 0.49. This moderate consistency is prone to lead to grading errors. It directly affects the selection of treatment plans (Reitzen et al., 2009). Although the Sunnybrook Scale enhances the objectivity of assessment through video recording, a single operation takes up to 15 min, and its application is limited in high-traffic scenarios in outpatient clinics (Cabrol et al., 2021). Secondly, the operational complexity of neuroelectrophysiological examination poses a significant obstacle. Electromyography (EMG) detection requires professional technicians to precisely locate the facial nerve branches. A single examination takes 30 to 45 min, and 15% of patients refuse to repeat the examination due to pain caused by the needled electrode (Holze et al., 2022). Furthermore, the contradiction between the cost and efficiency of imaging assessment is prominent. Although high-resolution CT of the temporal bone can show bony structural damage, the missed diagnosis rate of early lesions such as neuroedema is as high as 35% (Jun et al., 2012). Although 3.0 T MRI can directly observe the course of the facial nerve, the cost of a single examination is high, and the imaging of the temporal bone segment often leads to a decrease in resolution due to motion artifacts. These limitations jointly lead to systemic challenges such as delayed diagnosis, lagging therapeutic effect evaluation, and uneven allocation of medical resources in the diagnosis and treatment of FP. There is an urgent need for new assessment techniques to break through the existing bottlenecks.

Intelligent sensing technology builds a closed loop system of biological signal acquisition processing feedback by integrating multimodal sensors, edge computing and wireless communication modules. Its *μ* V-level signal parsing accuracy and sub-millimeter-level motion capture capability achieve ultra-fine perception of facial neuromuscular activities. Combined with real-time data stream analysis technology, it has completely broken through the bottleneck of accuracy and efficiency of traditional assessment methods (Walker et al., 2022). At the clinical application level, the graph neural network model has increased the accuracy rate of FP grading diagnosis to 95.1% by analyzing the spatio-temporal characteristics of 468 facial key points, and the diagnosis time has been compressed from 15 min of the traditional scale to within 0.5 s (Liu et al., 2020). The three-dimensional (3D) motion capture system can continuously record facial motion trajectories for 72 h, revealing a 32% incidence of associated motion that was missed in traditional evaluations, providing a key basis for formulating intervention plans (Lou et al., 2021). Individualized treatment plans based on intelligent sensor data have shortened the average recovery period of patients by 40% and reduced the recurrence rate by 27% (Guarin et al., 2020). This technology is driving the FP diagnosis and treatment model to make a paradigm leap from experience-dependent to data-driven, providing a revolutionary tool for the construction of a precision medical system.

Currently, the research gaps in the field of facial paralysis mainly lie in the following three aspects. Firstly, there is insufficient integration of technologies. Most studies only focus on a single modality, such as relying solely on surface electromyography (sEMG) or 3D motion capture, failing to fully utilize the advantages of multi-modal data fusion. Secondly, clinical translation is lagging behind: the existing models mostly remain at the laboratory validation stage and lack validation in actual scenarios such as the formulation of personalized rehabilitation plans and dynamic efficacy assessment. Thirdly, cross-disciplinary collaboration is weak: the interdisciplinary research between medicine, engineering, and data science is insufficient, resulting in a disconnection between technical solutions and clinical needs. The main research focus of this paper is to analyze the clinical value and limitations of three technical directions: sEMG, 3D motion capture, and artificial intelligence (AI) assisted analysis. This aims to provide a theoretical basis for the clinical application of intelligent sensing technology in FP, and also to indicate the direction for future interdisciplinary collaboration and technical optimization.

## 2 Principles and classification of intelligent sensing technology

### 2.1 sEMG technology

sEMG technology provides a non-invasive and highly time-resolution detection method for the assessment of facial nerve function by capturing the bioelectrical activities generated during muscle contraction. Its working principle is based on the spatiotemporal superposition effect of muscle fiber action potentials. When the muscles innervated by the facial nerve (such as the zygomatic major muscle and orbicularis oculi muscle) contract, the action potentials generated by the motor units are conducted along the muscle fibers, forming a detectable potential difference on the skin surface. The sEMG electrode extracts these weak signals through differential amplification technology. After filtering to remove noise, they are transformed into time-frequency domain parameters reflecting the degree of muscle activation.

The core advantage of this technology lies in its direct association with the functional state of the facial nerve muscles. Studies have shown that the root mean square value of sEMG signals on the affected side of patients with FP is 37–62% lower than that on the healthy side, and it is significantly negatively correlated with the House-Brackmann classification (*r* = −0.84, *p* < 0.001) (Cui et al., 2020). In addition, sEMG spectrum analysis can reveal the pathological characteristics of neuropathy: The median frequency of patients with acute FP decreased by 21% compared with the healthy population, while patients in the chronic phase showed progressive loss of high-frequency components (>200 Hz). These indicators provide quantitative basis for differentiating the nature of the lesion, such as nerve disuse vs. axonal rupture (Franz et al., 2024). In clinical applications, sEMG technology has broken through the positioning of a single diagnostic tool. By synchronically collecting the electromyographic activities of the bilateral masseter muscles and frontal muscles, the facial symmetry index can be quantified, providing precise target localization for botulinum toxin injection therapy combined with exercise. Furthermore, combined with 3D motion capture data, sEMG signals can analyze the neural control strategies of complex expression movements, such as the cooperative activation mode of the zygomatic major muscle and the orbicularis oculi muscle in the smiling movement, providing biofeedback for the expression reconstruction training of patients with FP (Petrides et al., 2023).

sEMG collects the summed potentials of superficial muscle groups through surface electrodes, with a low spatial resolution and being susceptible to interference from factors such as subcutaneous fat. However, it can collect dynamic electromyographic signals in real time, and its accuracy depends on the signal processing algorithm and electrode layout. It is mainly used in auxiliary fields such as rehabilitation assessment. Needle EMG inserts needle electrodes into the muscle, achieving a spatial resolution of millimeters, which can precisely locate deep or small area muscle lesions and accurately determine the nature of nerve-muscle lesions. It is recognized as the “gold standard” for the diagnosis of neuromuscular diseases, but it cannot directly monitor dynamic functions. The differences between sEMG and needle EMG in clinical practical applications are shown in Table 1.

**Table 1:** Comparison of advantages and disadvantages between sEMG and needle EMG.

Dimension	sEMG	Needle EMG
Intrusive	Non-invasive, skin surface collection, high patient acceptance.	Invasive, requires needle insertion into the muscle, may cause temporary pain or bleeding, and requires high skill from the operator.
Signal stability	Susceptible to skin impedance, electrode displacement, and environmental noise interference. Strict temperature control is required (such as avoiding sweating).	The signals are directly derived from within the muscle, are less affected by external factors, but may decrease in reliability when re-examined due to changes in needle position.
Detection range	Can cover the entire layer of superficial muscles, suitable for evaluating muscle coordination (such as gait analysis).	It can only detect a small area of muscle around the needle tip and requires multiple needle insertions at different points to assess the function of the entire muscle
Dynamic monitoring	Supports real-time collection of electromyographic signals during movement, suitable for dynamic functional assessment (such as analysis of football shooting movements).	Only applicable in rest or controllable contraction states, unable to directly monitor dynamic functions.
Clinical applicability	Rehabilitation medicine (such as muscle strength assessment for hemiplegic patients), sports science (such as determination of exercise fatigue), ergonomics (such as posture optimization).	Neurology (such as peripheral neuropathy, motor neuron disease), orthopedics (such as nerve root compression), neuromuscular junction diseases (such as myasthenia gravis).
Equipment cost and operation	The equipment is portable and easy to operate, but requires professional signal processing software support.	The equipment is expensive (such as electromyography machines priced from tens of thousands to hundreds of thousands), requires professional training and takes a long time to operate (each examination takes approximately 0.5 to 2 h).

### 2.2 3D motion capture technology

3D motion capture technology tracks the movement trajectories of facial marker points with sub-millimeter accuracy through optical or inertial sensor systems, and builds digital expression models. The optical system uses high-speed infrared cameras to capture the position changes of reflective marking points, with a sampling frequency of up to 200 Hz and a spatial resolution better than 0.1 mm. The inertial measurement unit directly measures the motion parameters of facial tissues through a miniature gyroscope and an accelerometer. The multimodal fusion system developed by the Mayo Clinic synchronously deploys 7 optical marker points and 4 distributed inertial measurement units, and combines the Kalman filtering algorithm to achieve spatio-temporal alignment of data, keeping the motion trajectory reconstruction error within 0.3 mm (Nguyen et al., 2022).

This technology reveals the functional characteristics of facial expression muscles by quantifying parameters such as displacement, velocity, acceleration and joint Angle. Clinical studies have shown that in the smiling movement of patients with FP, the angular displacement of the affected side is reduced by 41% compared with the healthy side, the peak speed of the blinking movement decreases by 57%, and the contraction acceleration of the zygomatic major muscle is significantly correlated with the conduction velocity of the facial nerve (Zhu et al., 2023). The facial nerve function map constructed based on kinematic parameters can dynamically evaluate the movement patterns of 12 basic expressions and discover minor functional disorders that cannot be recognized by traditional scales. In addition, this technology provides a quantitative basis for individualized rehabilitation programs. By correcting abnormal movement patterns through biofeedback training and combining it with the immersive rehabilitation system developed with VR technology, the naturalness score of patients’ expressions has been significantly improved, and the treatment compliance has increased to 89% (Qidwai et al., 2019).

### 2.3 AI assisted analysis technology

AI has significantly enhanced the accuracy and objectivity of FP diagnosis and treatment through deep learning models and multimodal data fusion technology. In the field of image and signal processing, convolutional neural networks demonstrate powerful feature extraction capabilities. For example, the FaceNet model improved based on the ResNet-50 architecture achieves an accuracy rate of 95.1% in the classification diagnosis of FP by introducing an attention mechanism to focus on key facial areas, and the diagnosis time is compressed to within 0.5 s (Feng et al., 2025). Recurrent neural networks excel at handling temporal biological signals, such as sEMG data stream analysis. Their Long Short-Term memory network structure can capture the temporal dependence of muscle activation patterns, increasing the accuracy of conjugated motion prediction to 89% (Zhuang et al., 2021).

Generative Adversarial Network (GAN) has opened up a new path for the diagnosis and treatment of FP. This technology can synthesize realistic facial expression images through adversarial training between the generator and the discriminator. For example, the Cycle GAN model generates a “virtual repair” image of the affected side expression by learning the expression mapping relationship between healthy people and patients with FP, enabling doctors to visually compare the symmetry of expressions before and after treatment. In more advanced applications, conditional GAN can dynamically generate corresponding expressions based on sEMG signals, providing a visual template for biofeedback training for patients with complete FP (DeBord et al., 2023). Furthermore, GAN has demonstrated value in data augmentation. By generating diverse pathological expression samples, it has expanded the training dataset size of deep learning models by 10 times, alleviating the bottleneck of scarce medical image annotation data.

The multimodal data fusion algorithm has further unleashed the clinical potential of AI. Feature-level fusion integrates sEMG spectral features, 3D motion trajectory parameters and patient self-rating scale data through deep neural networks to construct a joint feature space. Experiments show that the AUC value of the fused feature vector in differentiating central and peripheral FP reaches 0.94, which is 21% higher than that of a single mode. Decision-level fusion adopts Bayesian networks or D-S evidence theory to integrate the results of different evaluation tools. For example, by combining the sEMG signal improvement rate, motion capture parameters and the prediction results of the AI model, a probability map of facial nerve function recovery can be generated, providing a quantitative basis for the formulation of individualized rehabilitation plans. This multi-dimensional data fusion strategy enables the diagnosis and treatment of FP to shift from the assessment of a single indicator to comprehensive decision-making based on biomechanics, electrophysiology and clinical phenotypes.

Although sEMG technology can reflect muscle activity, the signals are prone to interference and have limited spatial resolution. 3D motion capture technology can accurately record movements, but the data processing is complex and the cost is high. AI assisted analysis technology relies on high-quality data and the models have poor interpretability. A single technology is difficult to comprehensively and accurately solve complex problems in practical applications. The integration of sEMG, 3D motion capture, and AI assisted analysis technology significantly enhances the accuracy, efficiency, and personalization level of facial paralysis recognition and assessment. Through the collaborative effect of electrophysiological signals, spatial motion data, and intelligent algorithms, the diagnostic accuracy of traditional methods (single sEMG or 3D Motion Capture) ranges from 70 to 80%, while with the integration of AI, it has improved to 95.1% (an increase of 25.1 percentage points). Combined with this technology, the false alarm rate of automatic assessment of facial paralysis grades can be reduced from 8 to 2%, the detection time can be shortened from several minutes in traditional methods to within 0.5 s (a speedup of approximately 90%), meeting the real-time requirements of clinical practice. Moreover, combined with this technology, the anxiety score of patients can be reduced by 37%, the efficiency of monitoring the rehabilitation progress can be increased by 50%, and personalized plans can reduce the repetitive work of doctors.

Previous studies on diseases such as FP or human movement often focused on a single aspect of assessment, such as only paying attention to muscle electrical activity or movement trajectories. The combination of these three techniques enables a comprehensive assessment from multiple dimensions, including muscle electrical activity, movement learning, disease diagnosis, and treatment effect evaluation. Moreover, by integrating these three techniques, individual differences such as muscle characteristics and movement patterns of patients can be fully considered, allowing for the formulation of personalized rehabilitation plans for each patient. Most importantly, the combination of these three techniques supports real-time data collection and analysis, enabling real-time tracking of disease progression and movement effects, and providing a basis for timely adjustment of treatment plans.

## 3 Application of intelligent sensing technology in FP recognition

### 3.1 Early diagnosis of FP based on sEMG

sEMG technology provides highly sensitive biomarkers for the early diagnosis of FP by capturing the bioelectrical activities of facial muscles. Taking the Biomask system as an example, this wearable device integrates an 8-channel flexible microneedle electrode array and an infrared temperature sensor, which can synchronously collect electromyographic signals and local blood perfusion data of key muscle groups such as the zygomatic major muscle and orbicularis oculi muscle (Cui et al., 2021). During the acute phase of Bell’s FP (within 72 h of onset), the Biomask system analyzed the time-frequency characteristics of sEMG signals through machine learning algorithms and successfully advanced the diagnostic window to 6.3 h after the appearance of symptoms, which was 52 h shorter than the traditional clinical assessment (Ryu et al., 2018).

The analysis of signal characteristics reveals the dynamic evolution law of facial nerve injury. The sEMG spectrum of patients with acute FP shows a “low-frequency migration” phenomenon. The median frequency is 21% lower than that of the healthy population, and it is strongly negatively correlated with the results of the facial nerve excitability test (*r* = −0.82). The combined time-frequency analysis further revealed that with the progression of the disease course, the high-frequency components (>200 Hz) of the electromyographic signal were progressively lost, and this change was particularly significant in patients with complete FP. Compared with traditional needle EMG, the Biomask system uses non-invasive microneedle electrodes, avoiding muscle twitch artifacts caused by acupuncture pain and improving the stability of the signal baseline by 67% (Demeco et al., 2021). In addition, its portable design supports home dynamic monitoring. In a multi-center study, continuous 72-h sEMG recording successfully captured the nocturnal associated motor episodes of 83% of patients, while the missed diagnosis rate of traditional single EMG detection was as high as 61% (Watts et al., 2020). This paradigm shift from “single-point assessment” to “continuous monitoring” has bought a valuable time window for the early intervention of FP.

### 3.2 The value of 3D motion capture in the classification of FP

3D motion capture technology quantifies the kinematic characteristics of facial expression muscles, providing an objective biomechanical basis for the clinical classification of FP. In the differentiation between central (supranuclear) and peripheral (subnuclear) FP, this technique reveals the differences in the trajectory characteristics of the two lesions: In the smiling movements of patients with central FP, the retention rate of the upward lift amplitude of the affected corner of the mouth can reach 67%, but there is a significant “synchronous contraction delay” phenomenon, which is related to the abnormal motor command conduction caused by cortical brainstem tract injury. However, patients with peripheral FP show a loss of symmetry in angular mouth displacement, and the contraction acceleration of the zygomatic major muscle is linearly correlated with the conduction velocity of the facial nerve (*r* = 0.76). By classifying the motion trajectory parameters through the Support Vector Machine algorithm, the automatic identification of two types of FP can be achieved, with an accuracy rate of 89.3%, which is 24% higher than that of the traditional neural localization diagnostic method (Tran et al., 2023).

This technology shows unique advantages in combined motion detection and postoperative functional assessment. The dynamic capture system can continuously record facial movement data for 72 h. Through spatio-temporal clustering analysis, it was found that 32% of patients with FP had minor conjunctival movements at rest. This abnormal pattern was not significantly correlated with the House-Brackmann scale classification, but indicated a poor prognosis. In the postoperative monitoring of facial nerve transplantation, the “functional recovery index” constructed by 3D motion capture combined with sEMG data can quantitatively evaluate the quality of nerve regeneration. Clinical studies have shown that for patients with this index >0.75 at 6 months after surgery, the improvement in the facial symmetry index was 2.3 times that of the low-index group, and the incidence of associated movement decreased by 58% (Zhao et al., 2020). In more advanced applications, the multimodal fusion of motion capture data and the fiber bundle tracking results of diffusion tensor imaging enables the visualization accuracy of the facial nerve regeneration path to reach 0.5 mm, providing precise anatomical navigation for secondary repair surgeries (Gupta et al., 2013).

### 3.3 AI assisted diagnosis model

AI has achieved the precision and individualization of FP diagnosis and treatment by constructing a deep learning framework. Models based on facial key point detection usually adopt a two-stage architecture: Firstly, 468 facial anatomical landmark points are located through the improved YOLOv7 algorithm, covering subtle areas such as the space between the eyebrows, nasolabial folds, and orbicularis oris muscle, with a detection accuracy of 0.8 pixels; Subsequently, the Graph Convolutional Network extracts the spatio-temporal features of the key points and fuses the dynamic information of the expression and action sequences. For instance, the FaceAI system achieved a differential accuracy rate of 96.4% for Bell’s FP and Hunt syndrome, which was 28% higher than that of experienced neurologists (Kong et al., 2024). The innovation of this model lies in the introduction of the self-attention mechanism, which enables the dynamic adjustment of the topological relationship weights between key points, thereby capturing minor functional disorders that cannot be identified by traditional scales.

In terms of performance evaluation, the FaceAI system demonstrates outstanding diagnostic efficiency. On the independent test set, its accuracy rate and recall rate reached 95.1 and 92.3%, respectively. It was significantly superior to the traditional House-Brackmann scale and Sunnybrook scale. Especially in the diagnosis of grade III - IV FP, the model synchronously optimizes the grading and localization tasks through a multi-task learning strategy, increasing the Kappa value to 0.89, which is much higher than the consistency among human evaluators (0.77). The ablation experiment confirmed that after fusing the sEMG spectral characteristics and motion capture parameters, the detection sensitivity of the model for linked band motion increased from 68 to 89%. In terms of clinical decision support, this system can generate individualized treatment suggestions. Based on the abnormal patterns of the patient’s facial movement trajectory, it automatically recommends the intensity of rehabilitation training, reducing the treatment plan formulation time by 73%. In a multicenter study in a tertiary hospital, the improvement in the facial symmetry index of patients who adopted AI suggestions was 1.9 times higher than that of the conventional treatment group, and the recurrence rate of associated movement was reduced by 54% (Zhang et al., 2024; Kimura et al., 2025).

In the current diagnosis and treatment of facial paralysis, sEMG and traditional needle-type EMG have the highest level of evidence strength (Level I), and should be used as the core diagnostic and monitoring tools; MRI and 3D motion capture technology need to be selected carefully in combination with clinical scenarios (Level II-III); although AI-assisted analysis has a promising future, it requires further verification (Level III). The key findings and evidence strength of each technology in the diagnosis and treatment of facial paralysis are shown in Table 2.

**Table 2:** Evidence-efficacy analysis of main assessment techniques for facial paralysis.

Technology	Research design	Sample size	Inclusion criteria	Exclusion criteria	Potential data bias analysis	Key findings	Level of evidence
sEMG	RCT	70 cases in the experimental group, 70 cases in the control group	Age 18–65 years; unilateral muscle lesion; no history of neurological diseases.	Diabetes/renal disease; history of hormone therapy within 3 months.	Small sample size may lead to type II errors; short-term follow-up may miss delayed effects.	The abnormal rate of muscle electrical activity detected by sEMG in patients with early facial paralysis was 82%, significantly higher than the 65% assessed by clinical signs (*p* < 0.05).	Level I
	Prospective cohort study	120 cases	Diagnosed with myopathy and abnormal EMG; voluntarily signed informed consent form.	Pregnancy/lactation; implanted electronic device.	Observational design has confounding factors (such as failure to control for differences in medication); prospective but non-randomized design may lead to selection bias.	The frequency domain analysis of sEMG (median frequency MF) showed a strong negative correlation with the House-Brackmann score 3 months after the onset (r = −0.78).	Level II
Traditional needle-type EMG	RCT	50 cases in the experimental group, 50 cases in the control group	Clinical diagnosis of neurogenic lesion; no coagulation dysfunction.	Severe cognitive impairment unable to cooperate; local infection lesion.	The small sample size (with only 50 cases in each group) may affect the statistical power; the details of the blinding process were not reported.	The nerve conduction velocity detected by needle EMG at the 3rd week after the onset was correlated with the degree of facial nerve edema (measured by MRI) at 0.85 (*p* < 0.01).	Level I
	Retrospective study	320 cases	The medical record is complete and conforms to the diagnostic criteria.	More than 20% of the key indicators are missing data.	The retrospective design has information bias; some clinical variables that were not recorded may have been missed.	The detection rate of self-generated potentials (fibrillation potentials) found by needle EMG in patients with Bell’s palsy (68%) was significantly lower than that in patients with Hunt syndrome (92%).	Level III
High-resolution MRI	Prospective multicenter study	256 cases	Suspected spinal cord/nerve root lesion; no MRI contraindications.	Severe spinal deformity; internal metal implants.	The standardization issue of multi-center data; the consistency verification of the scanning protocol was not reported.	MRI showed that when the diameter of the facial nerve at the junction with the cerebral aqueduct was >2 mm and the distance was <3 mm, the incidence of facial muscle spasm increased by 3.2 times (OR = 4.1, 95% CI 2.3–7.4).	Level II
	Case–control study	80 cases in the experimental group, 80 cases in the control group	Pathological confirmed case; gender/age-matched healthy control.	The control group has undiagnosed subclinical lesions.	The selection of cases may lead to deviations in diagnostic accuracy.	The diameter of the facial nerve measured by MRI was thicker by 1.8 ± 0.3 mm in the acute phase (< 7 days) compared to the healthy side, and reduced to 0.5 ± 0.2 mm in the recovery phase (> 3 months) (*p* < 0.001).	Level II
3D motion capture	RCT	30 cases in the experimental group, 30 cases in the control group	Patients with abnormal gait; no severe bone and joint deformity.	Recent surgical history; unable to complete test movements.	The extremely small sample size (n = 60 in total) results in low credibility of the results; the standardization of the motion capture environment is insufficient.	The asymmetry index of mouth corner deviation (ASI) detected by motion capture technology showed a consistency of 92% with the clinical score (House-Brackmann) in patients with severe facial paralysis (Kappa = 0.85).	Level I
	Cross-sectional study	150 cases	Community healthy adults; no motor dysfunction.	Professional athlete or long-term bedridden individual.	The cross-sectional design cannot determine causal relationships.	The motion capture technology revealed that 60% of the patients showed no obvious abnormalities under static expressions, but a movement sequence difference between the healthy side and the affected side occurred during dynamic smiling (> 0.2 s).	Level III
AI-assisted analysis	RCT	100 cases in the experimental group, 100 cases in the control group	The image data meets the quality standards; there are no artifacts or interference.	Motion artifacts exceeding 30% of the slices.	The consistency of manual annotation has not been evaluated.	The accuracy rate of predicting the probability of facial paralysis recovery by AI combined with sEMG data reached 88%, which was 15% higher than that of single sEMG analysis (*p* < 0.01).	Level I
	Retrospective cohort study	100 cases	The complete medical records comply with the input requirements of the algorithm.	The data format is incompatible.	The quality of historical data varies; the effect of time trends has not been addressed.	The sensitivity of the AI model for identifying rare causes of facial paralysis (such as Lyme disease) was 76%, and the specificity was 89%, but further verification is needed (due to high risk of dataset bias).	Level III

Level I evidence (direct recommendation): sEMG is used for early screening, needle EMG is used for locating nerve injuries, and AI-assisted analysis is used for prognosis prediction. Level II evidence (conditional recommendation): MRI is used to rule out tumors or vascular compression, motion capture technology is used for dynamic functional assessment. Level III evidence (cautious recommendation): Needle EMG distinguishes the cause, AI identifies rare causes.

## 4 Application of intelligent sensing technology in the assessment of FP

### 4.1 Quantitative evaluation system for therapeutic effect

Intelligent sensing technology, through multimodal data fusion and dynamic monitoring, has constructed a quantitative index system for the therapeutic effect evaluation of FP. The assessment system based on sEMG developed by Tsinghua University uses skin-friendly and breathable PU membrane electrodes to cover core muscle groups such as the frontal muscle, zygomatic major muscle, and orbicularis oculi muscle, and combines a wireless transmission module to collect signals in real time. This system accurately quantifies the differences in muscle activity between the affected side and the healthy side by analyzing the standard deviation of movement (MSD) and the correlation of signal energy. Clinical data show that the MSD of the affected side muscles in patients with FP is 41% lower than that of the healthy side, while the difference in bilateral MSD between healthy people is only 7%. The monitoring sensitivity of this system for treatment response reaches 92%, and it can capture the tiny electrophysiological changes in the early stage of nerve regeneration, providing a real-time basis for adjusting the treatment plan (Frigerio et al., 2015).

3D motion capture technology further enriches the dimensions of therapeutic effect evaluation. The multimodal system of the Mayo Clinic tracks facial movement trajectories with an accuracy of 0.3 mm through 7 optical marker points and 4 distributed IMUs. This system can extract parameters such as displacement, velocity, acceleration and joint Angle, and quantify the recovery of facial expression muscle function. For example, in the smiling movement of patients with FP, the displacement of the corner of the mouth on the affected side is 40% less than that on the healthy side, while after treatment, this indicator improves at a rate of 8% per week. Dynamic assessment also revealed associated movement patterns that could not be identified by traditional scales. For instance, 28% of patients experienced involuntary contractions of the nasolabial folds in the early stage of rehabilitation. The correction of such abnormal movement trajectories is closely related to long-term prognosis (Nguyen et al., 2022). The feasibility analysis of the main assessment plan for facial paralysis is shown in Table 3.

**Table 3:** Feasibility analysis of the main evaluation scheme for facial paralysis.

Technical type	Stage	Advantages	Limitation	Reusability	Clinical feasibility	Methodological limitations
sEMG	Diagnosis	Non-invasive, dynamic monitoring, suitable for early screening	The signal is affected by fat/muscle thickness, resulting in poor localization of deep injuries.	For children and patients with fear, it is the first choice. It should be combined with clinical judgment.	The grassroots institutions can be implemented, but training of the operators is necessary.	Signal analysis relies on algorithms and is sensitive to environmental interference.
	Treatment monitoring	Real-time monitoring of muscle activation sequence, guiding electrical stimulation training	It requires interpretation by professional physicians and has a low rate of long-term compliance.	Combined with needle-type EMG, the number of puncture points can be reduced.	It has good compatibility with rehabilitation training.	The cost of equipment limits its widespread adoption at the grassroots level.
	Prognosis assessment	Quantifying the muscle fatigue index, supporting home-based follow-up	A unified sampling standard is needed, and it is difficult to integrate data from different centers.	Combined with AI, the prediction accuracy can be improved.	Wireless devices improve compliance, but the dropout rate remains high.	The challenge of maintaining long-term data consistency.
Traditional needle-type EMG	Diagnosis	Gold standard: Quantitative assessment of nerve conduction velocity / CMAP amplitude	Invasive, pain risk, inability to conduct dynamic assessment	Identification of myogenic lesions cannot be replaced	Requires professional center operation, with low patient acceptance rate	Insufficient standardization of operations, subjective interpretation of results
	Treatment monitoring	Repeated detection of CMAP changes to evaluate nerve regeneration	Requires frequent punctures, causing strong discomfort for patients	Combining sEMG reduces the number of operations	Clinically mature but with poor experience	Dependence on the operator’s experience
	Prognosis assessment	Directly reflects the ability of nerve regeneration and predicts the recovery time	The accuracy of prediction by a single indicator is limited	It requires the combination of multimodal data	High clinical recognition but low usage frequency	Difficulties in implementing long-term follow-up
High-resolution MRI	Diagnosis	Clearly display the anatomical relationship of the facial nerve, diagnose vascular/tumor lesions	The early diagnosis of facial neuritis has low value and high cost.	Central facial paralysis is the first choice, and it cannot be replaced by EMG	Multidisciplinary collaboration is required, and the examination process takes time.	Spatial resolution contradicts scan time
	Treatment monitoring	Dynamically assess the position of blood vessels and nerves, guide the timing of surgery	The risk of frequent scans and the cost issue.	Realize personalized treatment adjustment through AI integration	This procedure is essential but has limited application scenarios.	There is no unified standard for dynamic monitoring
	Prognosis assessment	Quantify the degree of facial nerve edema, assist in the prognosis model	The radiation concern for long-term follow-up (although there is no ionizing radiation).	It needs to be combined with EMG data	Its scientific research value is greater than its routine clinical use.	There are significant differences in equipment accessibility
3D motion capture	Diagnosis	Quantify facial movement asymmetry, objectively assess functional deficits	It is necessary to actively cooperate. Patients with severe paralysis are restricted.	Alternative solutions for children/cognitive-impaired patients	Improving accuracy by integrating rating systems (such as House-Brackmann)	The landmark point positioning algorithm needs to be optimized.
	Treatment monitoring	Provide biofeedback for rehabilitation training, adjust movement patterns	The portability of the equipment is poor, making it difficult to use at home.	Real-time correction through sEMG integration	Has good application prospects in rehabilitation institutions	The cross-race/age database is missing.
	Prognosis assessment	Record long-term movement trajectories, evaluate the quality of functional recovery	The loss of patients leads to incomplete data.	A need to design incentive mechanisms to enhance compliance	Has great potential after wireless transformation	Data privacy and storage challenges.
AI-assisted analysis	Diagnosis	Automatically identify subtle asymmetries, with an early warning accuracy rate of over 85%	Dependence on the quality of training data, the recognition of rare diseases is weak	Substitute expert diagnosis with grassroots institutions	The model performance needs to be verified regularly.	The black-box algorithms have low clinical acceptance.
	Treatment monitoring	Dynamically adjust the electrical stimulation parameters to achieve personalized rehabilitation	It is necessary to establish a specific model for facial paralysis, and the transfer of a general architecture is difficult	Improve effectiveness by integrating sEMG/MRI data	The standardized potential of electrical stimulation therapy.	The real-time processing requires high hardware capabilities.
	Prognosis assessment	Integrate multimodal data to build a predictive model, with an accuracy rate increase of 20%	The standardization of cross-device data is lacking, and a collaborative framework has not been established	Promote the construction of multi-center databases	The progress in the research end is faster than that in clinical transformation.	There are obstacles in terms of ethical review and data sharing.
Multimodal fusion (sEMG+3D + AI)	Diagnosis	Synchronously analyze the electrical signals and the movement trajectories, and identify complex injuries (such as nerve compression + muscle atrophy)	It is necessary to address the time synchronization error among multiple devices (<1 ms), and the algorithm complexity is high.	It can replace a single technical combination in the identification of complex causes.	Multidisciplinary team collaboration is required. The new generation of equipment has achieved hardware-level synchronization.	Dependence on the quality of cross-modal data annotation, and insufficient training data for rare disease types.
	Treatment monitoring	Real-time adjust the parameters of electrical stimulation (frequency/pulse width) and the intensity of exercise training	It requires edge computing hardware to support real-time analysis, and the equipment cost will increase by 3–5 times.	It cannot replace personalized rehabilitation plans, but can be simplified to portable solutions (such as mobile phone AI + wireless sEMG).	Clinical trials show that patient acceptance has increased by 40%, but professional remote monitoring is necessary.	The cross-device communication protocol is not unified.
	Prognosis assessment	Build a dynamic prediction model (movement range + fatigue index), with an accuracy rate that is 35% higher than that of a single modality	It is necessary to accumulate long-term follow-up data.	It is not replaceable in research scenarios, and the clinical routine application needs to simplify the indicators.	The construction of cross-center databases promotes standardization, but the time for ethical review has increased.	The model’s interpretability is poor, and clinicians have low trust in the “black box” results.

### 4.2 Multi-dimensional evaluation and long-term efficacy prediction

Combined with the mixed model of patient self-rating scales, a comprehensive assessment from physiological signals to quality of life has been achieved. A multicenter study from Busan National University in South Korea showed that the integrated traditional Chinese and Western medicine treatment group improved the EQ-5D-5L health score by 15% compared with the conventional treatment group, and the improvement rate of facial sEMG signals was significantly positively correlated with the quality of life score (Goo et al., 2025). This correlation continued to strengthen during the 6-month follow-up, suggesting that the recovery of physiological functions is the basis for the improvement of quality of life. Furthermore, there is a strong correlation between the motion capture parameters and the social function dimension in the FACIAL-QOL scale. For every 10% increase in facial motion symmetry, the patient’s social confidence score increases by 0.8 points accordingly.

Long-term follow-up studies have verified the prognostic predictive value of intelligent sensing technology. A team conducted a 6-month follow-up on 85 patients with FP and found that the correlation of sEMG signal energy increased from 0.51 at the beginning of treatment to 0.73, and this index was linearly related to the recovery of facial nerve conduction velocity. Motion capture data show that the symmetry index of the facial movement trajectory of patients improves at a rate of 12% per month. For patients with an symmetry index of the facial>0.85 after 3 months of treatment, the risk of recurrence is reduced by 67%. What is more worthy of attention is that the AI model has constructed an efficacy trend prediction algorithm by integrating sEMG spectral features, motion capture parameters and patients’ self-evaluation data. The prediction error of this model for the rehabilitation period of patients with complete FP is only ±1.2 weeks, which is three times more accurate than the traditional empirical judgment method, providing a scientific basis for the dynamic optimization of individualized treatment plans (Miller et al., 2021). The clinical diagnosis and treatment of facial paralysis require the integration of multimodal technologies to achieve precise diagnosis, dynamic monitoring, and personalized prognosis assessment (Kafle and Thakur, 2021; Shi et al., 2024; Kishimoto-Urata et al., 2023). The analysis of the current status and future development direction of clinical diagnosis and treatment techniques for facial paralysis is presented in Table 4.

**Table 4:** Current status and future development direction of clinical diagnosis and treatment techniques for FP.

Diagnosis and treatment phase	The current treatment pathway	Key technical limitations	Research gap	Future recommendation direction
Diagnosis stage	Clinical assessment: House-Brackmann scale, Sunnybrook scoring system. Imaging examination: MRI (to rule out tumor/vessel compression). Electrophysiological examination: Needle EMG (gold standard), sEMG (screening).	The needle-type EMG is highly invasive and has poor patient compliance. sEMG is not accurate in locating deep nerve injuries. MRI has limited value in the early diagnosis of acute facial neuritis.	Diagnostic accuracy of the AI model. Fusion diagnostic criteria for multimodal data (sEMG + 3D motion capture).	Short-term: Promote portable sEMG + 3D + AI-assisted screening, covering primary medical care. Long-term: Develop non-invasive neural signal detection technology.
Treatment monitoring stage	Drug therapy: Glucocorticoids, antiviral drugs. Physical therapy: Electrical stimulation, facial muscle training. Surgical treatment: Microvascular decompression (for vascular compression-induced facial paralysis).	The traditional electrical stimulation parameters are based on experience and lack personalized adjustment. Motion capture requires active cooperation from the patient, and its application is limited for severely paralyzed individuals. The cost of MRI dynamic monitoring is high.	AI-driven dynamic optimization algorithm for electrical stimulation parameters. Strategies for improving patient compliance during long-term treatment (such as home monitoring devices).	Mid-term: Establish an “sEMG + AI” closed-loop electrical stimulation system to enable real-time adjustment of treatment parameters. Long-term: Develop implantable flexible electrodes to achieve synchronous collection of neural-muscular signals.
Prognosis assessment stage	Clinical follow-up: Regular re-evaluation of the House-Brackmann score. Electrophysiological re-examination: Needle EMG is used to assess nerve regeneration. Imaging re-examination: MRI is employed to observe the resolution of nerve edema.	The accuracy of prediction based on a single indicator (such as CMAP amplitude) is insufficient. The rate of patient attrition during long-term follow-up is high. There is a lack of a cross-center data sharing platform.	Validation of the multimodal prognostic prediction model (electrophysiology + 3D motion capture + AI). Effective collection and analysis of patients’ home rehabilitation data.	Short-term: Design a patient incentive follow-up mechanism (such as exchanging health points for medical treatment discounts). Long-term: Build a national-level facial paralysis diagnosis and treatment database and promote cross-center validation of AI models.
Technical integration requirements	Multidisciplinary collaboration: Joint diagnosis and treatment by neurology department, otolaryngology department and rehabilitation department.	The data formats of the equipment are not uniform (such as the sampling frequency of sEMG and the slice thickness of MRI scans). There is a lack of a cross-modal AI analysis framework.	A multimodal data fusion standard specifically for facial paralysis. The compatibility between portable devices and large-scale equipment in hospitals.	Mid-term: Develop standardized guidelines for the diagnosis and treatment of facial paralysis. Long-term: Develop an AI platform for “diagnosis - treatment - follow-up” integration.

## 5 Discussion

The combination of sEMG, 3D motion capture, and AI assisted analysis technology has opened up new paths for the precise diagnosis, personalized treatment, and rehabilitation assessment of FP. However, we have identified the following limitations in recent studies. The facial paralysis data mostly come from a single center, lacking unified collection standards, and there is a scarcity of public datasets, which affects the generalization of the models. Most related studies focus on adult patients, with insufficient coverage of children, the elderly, or samples from different ethnic groups, which may lead to model bias. Most studies concentrate on mild-to-moderate facial paralysis, and the validation for severe paralysis or post-lesion patients is limited. The current research has limitations in data diversity, validation scope, and technical universality. To enhance the model’s generalizability and clinical applicability, multi-center collaboration, standardized data collection, and optimization for special populations are needed. Future research can be deepened in the following directions.

### 5.1 Multi-modal data fusion and algorithm optimization

By integrating the millimeter-level accuracy of 3D motion capture, a “holographic map” of facial muscle activity can be constructed. For example, by covering key muscle groups such as the frontal muscles and orbicularis oculi with dense electrode arrays, simultaneous capture of muscle electrical activity and movement trajectories can be achieved, solving the problems of signal interference and blurred positioning in traditional methods (Zhu et al., 2022). Using deep learning algorithms (such as convolutional neural networks, graph neural networks) to perform real-time fusion analysis of multi-modal data and establish a dynamic model of the facial nerve-muscle system. For example, by training AI to identify differences in facial movement patterns between healthy individuals and patients with facial paralysis, automatic classification of the causes (such as viral infection, trauma, or tumor compression) can be achieved (Petrides et al., 2022).

### 5.2 Precise diagnosis and early warning

By integrating facial expression analysis technology, through AI monitoring of subtle changes such as blink frequency and symmetry of the corners of the mouth, a facial paralysis risk prediction model is constructed (Gaber et al., 2022). The combination of sEMG and nerve conduction velocity detection is utilized to analyze the electrophysiological characteristics of the facial nerve in real time. AI can identify early signals of nerve damage by comparing normal and abnormal EMG data, thus securing the golden time for treatment (Zhang et al., 2022). In the future, environmental factors (such as cold stimulation) and patient medical history can be further integrated to improve the accuracy of warning.

### 5.3 Personalized rehabilitation and biofeedback therapy

An AI-based biofeedback training platform is developed. Through 3D motion capture, real-time monitoring of patients’ facial movements is conducted, and the training intensity is adjusted in combination with sEMG data (Fattah et al., 2014). Studies have shown that high-resolution sEMG can quantify the effect of biofeedback training on facial muscle coordination. In the future, combined with virtual reality technology, immersive rehabilitation scenarios can be designed to enhance patient engagement (Dusseldorp et al., 2018). Exploring the combination of sEMG with ultrasound imaging, brain-computer interface (BCI), and achieving more precise neuromuscular control is also possible. For example, through ultrasound imaging to verify the source of muscle activity recorded by sEMG, combined with BCI technology, help severely affected patients recover facial expression functions.

### 5.4 Clinical application and standardization construction

Promote the miniaturization and wirelessization of sEMG and 3D motion capture technology, and develop rehabilitation monitoring devices suitable for home use. For instance, the combination of flexible electrode patches and smartphone apps enables real-time upload of patients’ daily rehabilitation data, facilitating remote adjustment of treatment plans by doctors (Zimmermann et al., 2019). Collaborate with medical institutions, research institutes, and enterprises to establish technical specifications for facial paralysis assessment and efficacy evaluation standards. For example, clarify operational details such as sEMG electrode layout and 3D motion capture marker point settings, to enhance the comparability of research results and the reliability of clinical application (Rao et al., 2025). In the future, with continuous technological breakthroughs, this multimodal integration solution is expected to achieve the “precise diagnosis - personalized treatment - dynamic assessment” full process management of facial paralysis, significantly improving patients’ quality of life and providing a new technical paradigm for the field of neurorehabilitation.
